# Induction of a common microglia gene expression signature by aging and neurodegenerative conditions: a co-expression meta-analysis

**DOI:** 10.1186/s40478-015-0203-5

**Published:** 2015-05-23

**Authors:** Inge R Holtman, Divya D Raj, Jeremy A Miller, Wandert Schaafsma, Zhuoran Yin, Nieske Brouwer, Paul D Wes, Thomas Möller, Marie Orre, Willem Kamphuis, Elly M Hol, Erik W G M Boddeke, Bart J L Eggen

**Affiliations:** Department of Neuroscience, section Medical Physiology, University of Groningen, University Medical Center Groningen, Groningen, The Netherlands; Allen Institute for Brain Science, Seattle, USA; Lundbeck Research USA, Paramus, New Jersey USA; Department of Astrocyte Biology and Neurodegeneration, Netherlands Institute for Neuroscience, Institute of the Royal Netherlands Academy of Arts and Sciences (KNAW), Amsterdam, The Netherlands; Department of Translational Neuroscience, Brain Center Rudolf Magnus, University Medical Center Utrecht, Utrecht, The Netherlands; Swammerdam Institute for Life Sciences, Center for Neuroscience, University of Amsterdam, Amsterdam, The Netherlands

## Abstract

**Introduction:**

Microglia are tissue macrophages of the central nervous system that monitor brain homeostasis and react upon neuronal damage and stress. Aging and neurodegeneration induce a hypersensitive, pro-inflammatory phenotype, referred to as *primed* microglia. To determine the gene expression signature of priming, the transcriptomes of microglia in aging, Alzheimer’s disease (AD), and amyotrophic lateral sclerosis (ALS) mouse models were compared using Weighted Gene Co-expression Network Analysis (WGCNA).

**Results:**

A highly consistent consensus transcriptional profile of up-regulated genes was identified, which prominently differed from the *acute* inflammatory gene network induced by lipopolysaccharide (LPS). Where the *acute* inflammatory network was significantly enriched for NF-κB signaling, the *primed* microglia profile contained key features related to phagosome, lysosome, antigen presentation, and AD signaling. In addition, specific signatures for aging, AD, and ALS were identified.

**Conclusion:**

Microglia priming induces a highly conserved transcriptional signature with aging- and disease-specific aspects.

**Electronic supplementary material:**

The online version of this article (doi:10.1186/s40478-015-0203-5) contains supplementary material, which is available to authorized users.

## Introduction

Neuroinflammation plays an important role in the progression of neurodegenerative diseases, with a prominent role for microglia [1-5]. Microglia are the primary innate immune cells of the brain and the first to respond to a variety of stimuli, like neuronal damage and infections, initially to restore homeostasis [6]. Upon activation, microglia release increased amounts of inflammatory cytokines, phagocytose cellular debris, and support tissue remodeling [6].

Microglia are versatile cells that, depending on environmental cues, are able to adopt different phenotypes but clear phenotypical identities have not been established. Microglia, like other cultured macrophages, are often classified into inflammatory (M1) and alternatively activated (M2) phenotypes [7,8], in which the M1 phenotype was originally induced using LPS or IFNγ stimulation, and the M2 phenotype using IL-4, IL-13 or IL-10.

In several neurodegenerative disorders and upon aging, chronic activation of microglia has been reported to induce a hypersensitive phenotype, often referred to as *primed* [9-11]. *Primed* microglia do not secrete high amounts of cytokines, but when triggered by pro-inflammatory stimuli, they become hyper-reactive, secreting large amounts of cytokines, chemokines, and other reactive molecules associated with neurotoxicity. We recently reported that microglia priming in a mouse model for accelerated aging was induced by an affected neuronal environment and not by intrinsic aging [12]. Although microglia priming is becoming a generally accepted concept [9], at present priming primarily is a functional definition and it is unclear whether microglia priming is a homogeneous phenotype with a specific transcriptional signature or a heterogenous phenotype with model-system specific transcriptional profiles and what the functional consequences of priming are.

In this study, these aspects were addressed by comparing the gene expression networks in pure cell populations of *primed* microglia that were isolated from mouse models for neurodegenerative disease and aging. The mouse models included are: **1)** aged mice; **2)** accelerated aging mice (Ercc1^∆/KO^), a DNA repair-deficient mouse model that displays features of accelerated aging [10]; **3)** APPswe/PS1dE9 (App-Ps1), a mouse model for Alzheimer’s disease, carrying transgenes for mutated Amyloid Precursor Protein and Presenilin-1 and **4)** a mouse model for Amyotrophic Lateral Sclerosis (Sod1^93A^, abbreviated as Sod1), a line carrying a mutation in the *SuperOxide Dismutase-1* gene, encoding an enzyme involved in free radical degradation, resulting in motor neuron degeneration in the spinal cord [4].

In addition, the microglia priming network was also analyzed using (unsorted) brain tissue expression data. The mouse models included are: **1)** aged mice; **2)** App-Ps1 mice; **3)** rTg4510, a mouse line expressing P301L mutant human tau [13,14]; **4)** an ME7 model of murine prion disease, associated with neuronal loss and microglial activation [15,16] (for an overview of mouse models and data sets used, see Additional file 1: Table S1).

Transcriptional profiles of microglia isolated from four mouse models of aging and disease and four brain tissue expression data sets were analyzed in parallel and compared using WGCNA [17]. In contrast to traditional differential gene expression analysis, co-expression network analysis does not regard genes as single entities, but incorporates the interrelation of genes to generate structures called modules. WGCNA has been reported to be a useful approach to integrate immunology with bioinformatics [18], and has been applied to evaluate common denominators in meta-analyses or disease models [1,19-21]. By raising the network to a power function, WGCNA results in a heterogeneous network dominated by a few highly connected nodes (hubs), which link the rest of the less connected nodes to the system [17]. These hub genes are likely control points or key genes that modulate the expression of the network-module and thereby are considered important for the observed phenotype [19,21,22]. In this paper, a WGCNA-based meta-analysis was applied to determine the transcriptional signature and hub genes of different microglia phenotypes: *primed*, age- and neurodegeneration-associated, and *acute* inflammatory.

## Materials and methods

### Microglia and brain tissue expression profiling

Pure *ex vivo* microglia populations were obtained by FACS sorting and RNA was isolated as recently described in [10,23]. Three microglia expression datasets were generated; 4 and 24 months old DBA/2 J and C57/SJL mice (Harlan, The Netherlands) were used. For *acute* LPS activated microglia, C57BL/6 mice (4 months, Harlan, The Netherlands) were i.p. injected with LPS (10 mg/kg) or PBS and microglia were isolated after 4 hr. RNA quantity and quality of the RNA samples was checked using the Experion RNA HighSense Analysis kit (BioRad, Cat.no. 700-7105), samples with high integrity (RIN > 7) were used for expression profiling. RNA was amplified with Nugen Ovation PicoSL WTA system (Cat nr. 3310-48), labeled with the Encore BiotinIL Module (Cat nr. 4210-48) and hybridized to Illumina MouseRef8 bead-chip microarrays. Raw data were generated using Illumina Genome studio.

rTg4510 mice carry a human P301L mutant tau transgene downstream of the tetracycline operon-responsive element (TRE), whose expression is driven by a second transgene expressing the tetracycline-controlled transactivator (tTA) under control of the Ca2+/calmodulin-dependent protein kinase II α (CaMKIIα) promoter. tTA constitutively induces tau expression via the TRE, but can be inactivated with doxycycline administration. Transgenic mice were bred at Taconic, Denmark. Mice expressing the tTA activator transgenes were maintained on a 126S6 background strain (Taconic) and mutant tau responder mice were maintained in the FVB/N background strain [14]. rTg4510 mice were perfused and sacrificed at 2, 4 , 6 and 8 months of age. RNA was isolated from brain tissue and hybridized to Illumina MouseWG6 bead-chip microarrays. All experiments were approved by the animal experimentation committees of the University of Groningen and the Royal Netherlands Academy for Arts and Sciences and are in accordance with the European Communities Council Directive #86/609 and the directives of the Danish National Committee on Animal Research Ethics. Previously published transcriptomes from pure microglia, brain tissue, and cultured and stimulated macrophages were included in our analysis, for detailed platform and, experimental design information see Additional file 1: Table S1 [15,24-28].

### Pre-processing of transcriptomes

Raw expression values were preprocessed using R and Bioconductor package Limma [29]. Samples with an average inter-sample correlation three standard deviations below the mean inter-sample correlation after normalization were filtered out and this procedure was repeated until all samples met the inclusion criteria. Quantile normalization was applied to the Illumina microarrays. To eliminate batch effects between both physiological aging datasets, the ComBat function was applied [30]. For Agilent array preprocessing, background correction was performed with an offset of 50 followed by Lowess within array normalization and Quantile between array normalization. Relative intensities were converted into expression values. The Affymetrix microarrays were preprocessed using the Expresso-function of R package Affy [31]. The parameters were set to RMA background correction and quantile normalization, with pm correct pmonly and a medianpolish. From the Sod1 RNA-sequencing dataset [4] the published RPKM-values were used, to which quantile normalization was applied to ensure that all samples have the same distribution in order to generate a more stable network.

### Select representative probes

Datasets from different platforms were made comparable at the level of gene symbols. The WGCNA collapseRows function was applied to calculate the representative gene expression for several probes, associated with a single gene [30]. The default method ‘MaxMean’ was used to select the row with the highest mean value. Similarly for the RNA-sequencing data several RefSeq accession numbers, associated on the same gene, were collapsed on gene symbols. Next, all gene symbols from the different platforms were intersected and only those genes that were present on all included platforms were used for further analysis.

### Parallel and consensus network formation

In all pure microglia datasets, genes with low variation or low connectivity were filtered out, resulting 7512 genes in the 5 parallel networks for the individual datasets, as described previously [17]. In the combined pure microglia and brain tissue analysis, no further filtering was applied, because less genes were present as more platforms were included, resulting in 9936 genes that were taken into this analysis. Subsequently, the topological overlap (TO) matrices from all five models were scaled such that the 95^th^ quantiles matched. A consensus TO matrix was calculated using the minimal value (pMin) for all gene pairs in any of the scaled TO matrices. From each of these six TO matrices, a dendrogram was generated by average linkage hierarchical clustering. Using the tree cut function, branches of highly co-expressed genes were grouped into modules. Only modules of a minimum size of 100 genes were considered for further analysis. Modules from the five model networks were defined using a hard-clustering approach, meaning that only genes directly clustered in the module were taken, and the module EigenGenes (ME) were calculated. For the consensus network, modules were defined using a soft-clustering approach, in which meta-q values and meta ME correlation thresholds were used to determine which genes were included (min correlation of 0.25 and min meta FDR-corrected q-value of 1E-8).

For each module, a Kruskall Wallis non parametric test, was used to assess differential expression of the ME with respect to aging or disease. Only modules with a p < 0.005 were considered to be differentially expressed, and were used for further analysis. A Fisher’s exact test was used to determine if the modules from the 5 datasets had a significant number of overlapping genes and these results were depicted as an overlap Heatmap. Modules were annotated by using WEB-based GEneSeT AnaLysis Toolkit (WEBGESTALT) to perform KEGG pathway and GO analysis [22,32]. The gene list that resulted from the intersection of the Illumina and Agilent arrays and Illumina Sequencing was used as the background list. To compare our modules to other gene expression studies WGCNA’s userListEnrichment function was used [33].

### Hub gene classification to compare different WGCNA core networks

The importance of a gene in a network module is determined by the strength of the correlation to the ModuleEigene, or module membership (kME) value [17]. The 35 genes with the highest (most significant) kME were taken from the networks to be analyzed. Module membership correlation thresholds were used to determine whether a gene is highly associated (i.e., a “hub” gene; FDR-q < 1.0E-11 for *primed* and *acute* FDR-p < 1E-7), moderately associated (below hub-gene association and FDR-p < 1.0E-2) or not associated (FDR-p > 1.0E-2) with a module. This strategy resulted in five clusters of genes: 2 clusters with hub genes significantly correlated with one and not the other network, 2 clusters with hub genes significantly correlated with one network and less significantly with the other dataset and a cluster containing hub genes strongly correlated to both networks.

### Gene set enrichment analysis: pre-ranked analysis

Systematic differences between two network modules were determined with gene set enrichment pre-ranked list analysis [33]. The 1000 most significantly module membership associated genes from either the *acute* and/or the *primed* networks were taken into the analysis, negative correlations were set to zero, and genes were ranked on strength of module membership to both networks. The difference in rank-values between consensus *primed* and *acute* was used as input for the analysis. GSEA pre-ranked list analysis was applied using a 1000 permutations.

### Quantitative RT-PCR and immunohistochemistry

Quantitative RT-PCR and immunohistochemistry were performed as described in [10]. See Additional file 2: Table S2 for primer information.

### Differential gene expression analysis

Differential gene expression was applied to the pure microglia datasets (see Additional file 3: Table S3 for these lists) as well as to datasets related to several *in vitro* stimulation conditions like LPS, IL-4, and IFNγ [27,34] which were used as genesets for UserListEnrichment. Differential gene expression was done using Limma [29] for microarray data and EdgeR for RNA-seq data [35].

## Results

### Aim and outline of the co-expression network analysis

**Figure 1:**
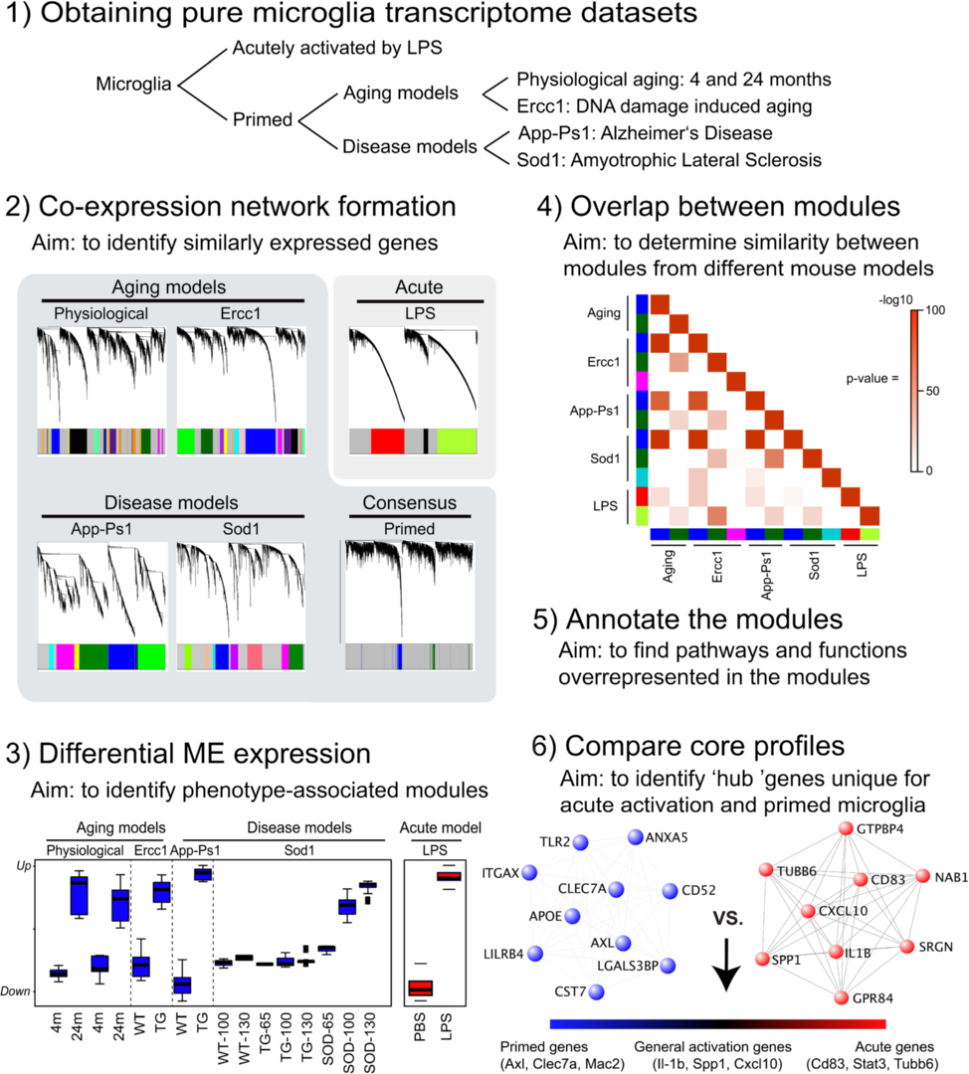
Outline of the WGCNA analysis. **Phase 1)** Obtaining pure microglia datasets. Transcriptome datasets were obtained from microglia of aged, accelerated aged, App-Ps1 transgenic (Alzheimer’s Disease model), Sod1 transgenic (Amyotrophic Lateral Sclerosis model), and i.p. LPS injected mice (*acute* activation). Each dataset contained its own control. **Phase 2)** Co-expression network formation. Co-expression networks were generated for 7512 genes of the indicated transcriptome datasets. Average linkage hierarchical clustering was applied to the topological overlap matrix and branches of highly correlating genes were formed, which were cut and assigned a color. *Primed* microglia networks were combined into a consensus network that represents the commonalities in the gene expression profiles of the individual *primed *microglia networks. **Phase 3)** Differential ME expression. For each module the Module Eigengene (ME) was calculated, which represents the expression profile of the module. A Kruskall Wallis between group test was applied to determine if ME values were significantly different between conditions, to find modules that were related to phenotype. The consensus primed microglia blue modules and acute red module are depicted as a box-plot containing the distribution of the ME values across the samples of each particular condition. **Phase 4)** Overlap between modules. The Fisher’s exact test was used to determine the significance of the overlap between modules from different model systems. **Phase 5)** Annotation of the modules. Modules were annotated using WebGestalt for GO and KEGG analysis. **Phase 6)** Comparison of core profiles. The correlation of each gene to the module EigenGene (kME) values was calculated for all genes in the analysis of the consensus blue *priming* module. These consensus *primed* microglia derived hub genes were subsequently compared to the acute activation network to find genes generally associated with activation, uniquely with *primed* microglia, or uniquely with *acute* LPS activation.

Microglia are versatile cells that adopt different activation states and become *primed* during aging and neuropathological conditions, but the transcriptional signature underlying the induction of this phenotype is yet unclear. To gain more insight in the microglia transcriptome during aging and neuropathological conditions, a WGCNA-based analysis workflow was set up consisting of several phases (Figure 1). In the first phase, expression profiles of pure populations of microglia from physiological aging, accelerated aging (Ercc1), disease mouse models for AD (App-Ps1) and ALS (Sod1) mice, and *acute* immune activated (i.p. injection with LPS) microglia were obtained and preprocessed in parallel (Figure 1, phase 1). Only genes that could be detected by all platforms were taken into account for further analysis. In the second phase, for each gene expression dataset, a network was created using WGCNA, resulting in modules that consist of branches of highly correlating genes (Figure 1, phase 2). In addition, the individual primed microglia networks were combined into a consensus network, which contains the commonalities of all four networks. For the *primed* microglia networks, module colors were initially randomly assigned and subsequently matched based on the number of overlapping genes. The aim of the third phase is to find modules related to the aging or neurodegenerative phenotype. Therefore, the Module Eigengene (ME) was calculated, which is the first principal component and represents the expression profile of the module. Differential ME expression was used to identify modules that associated with aging or neurodegeneration. In the fourth phase, the similarity between these response modules from different networks was determined with pair-wise comparisons. In the fifth phase, the modules were annotated using KEGG-pathway and Gene Ontology enrichment analysis, in order to obtain a better understanding of the implications of priming for microglia function. In the sixth phase, ‘hub’ genes were determined of the *acute* and *primed* microglia consensus networks by module membership (or kME) and compared. When microglia become *primed*, a strikingly similar transcriptional network (the consensus blue module) is induced which is distinct from the network induced by *acute* activation (LPS).

### Generation of co-expression networks and identification of modules related to phenotype

Expression profiles of pure microglia populations from different mouse models were used to generate co-expression networks. In these co-expression networks, we searched for WGCNA modules that were differentially expressed between conditions (i.e. young vs. aged, control vs. App-Ps1 etc.). Two classes of differentially expressed modules were identified; either up-regulated or down-regulated between conditions. The up-regulated modules are the blue modules in aged, Ercc1, App-Ps1, and Sod1 microglia, the red module in LPS-stimulated microglia as well as the Sod1-specific dark-turquoise module. The down-regulated modules are the dark-green modules in aged, Ercc1, App-Ps1, and Sod1 microglia, the magenta module in Ercc1, and the green-yellow module in LPS-stimulated microglia. For an overview of all modules and their relation to different conditions with their associated p-values see Additional file 4: Table S4.

To determine the (dis)similarities between these modules, a pair-wise comparison of all differentially expressed modules was performed. A highly significant overlap was observed between the 4 up-regulated blue modules (p-values ranging from 1.79E-79 to 1.62E-146) (Figure 1, phase 4) and the overlap of these blue modules with the *acute* LPS-induced red module was much less (p-values ranging from 2.44E-8 to 1.13E-29. These data indicate that aging and neurodegeneration induce a very similar up-regulated gene expression profile in microglia. For a pair-wise comparison between all modules identified in all mouse models, see Additional file 5: Figure S1.

The overlap between the down-regulated dark-green modules in aged, Ercc1, App-Ps1, and Sod1 microglia, as well as the overlap of these modules with the down-regulated green-yellow module of LPS-activated microglia, was less pronounced (p-values ranging from 1.67E-04 to 5.56E-65). No significant overlap of the Ercc1-specific magenta module with any other differentially expressed module was observed.

In order to address the observed overlap between the blue and dark-green modules in aged, Ercc1, App-Ps1, and Sod1 microglia, we generated a consensus network, consisting of co-expressed genes shared between the four individual datasets. This consensus network contained two modules: a blue and a dark-green module. The consensus blue module, consisting of 295 genes, is up-regulated and the consensus dark-green consensus module (205 genes) is down-regulated in aged, Ercc1, App-Ps1, and Sod1 microglia (these gene lists can be found in Additional file 6: Table S5).

### GO and KEGG annotation of modules related to phenotype

**Figure 2:**
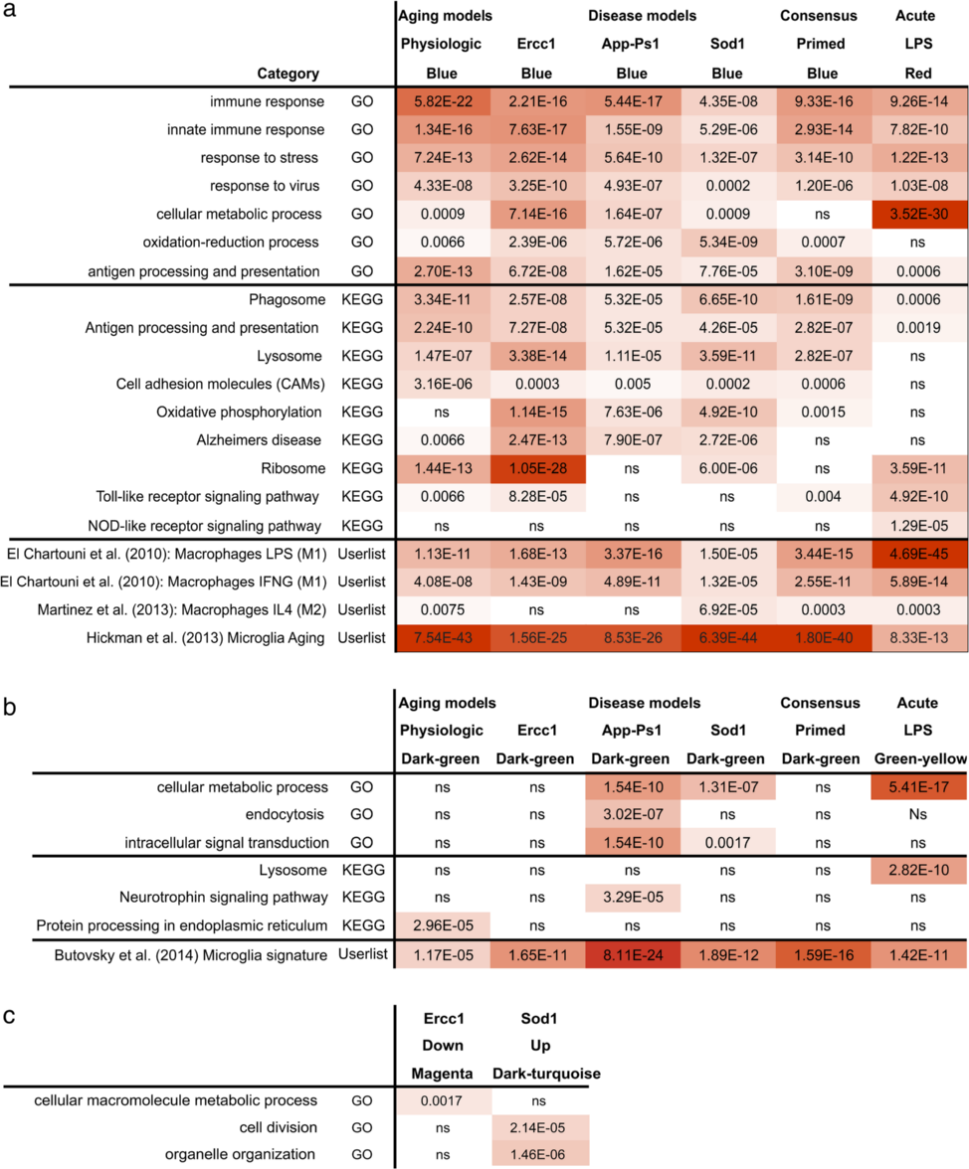
Annotation of the up- and down-regulated modules. **a)** The up-regulated *priming* and *acute* activation modules are distinct. The main up-regulated modules (blue modules for *priming* datasets and red for the *acute* dataset) were annotated with Webgestalt to determine significantly enriched KEGG-pathways and Gene Ontologies. These results are depicted with the multiple testing (FDR) corrected p-values. Using the UserListEnrichment function, significance was calculated for the overlap between these modules and gene sets significantly up-regulated in macrophages stimulated with IL-4, IFN? or LPS, and microglia-aging profile. For the UserlistEnrichment results Bonferroni multiple-testing p values are shown. **b)** KEGG-GO and UserlistEnrichment annotation of the down-regulated modules. The main down-regulated modules (dark-green modules for *priming* datasets and green-yellow for the *acute* dataset) were annotated with Webgestalt to determine significantly enriched KEGG-pathways and Gene Ontologies. These results were depicted as a table with multiple testing (FDR) corrected p-values. Using the UserListEnrichment function, significance was calculated for the overlap between these modules and the Butovsky microglia-signature [36]. For the UserlistEnrichment results Bonferroni multiple-testing p values are shown. **c)** KEGG-GO annotation of mouse model specific modules The Ercc1 down-regulated magenta module and ALS dark-turquoise up-regulated module were GO annotated, multi-testing (FDR) corrected p values are depicted.

The *primed* microglia blue modules (up-regulated in aged, Ercc1, App-Ps1, and Sod1 microglia) and the *acute* LPS-activated microglia red module (up-regulated in *acute* LPS activated microglia) were most strongly enriched for GOs “immune response” and “response to stress” and KEGG pathways significantly enriched in the priming blue modules were: “Alzheimer’s disease signaling”, “antigen-presentation”, “lysosome” and “phagosome”. The *acute* red module was most significantly enriched for the “ribosome”, “Toll-Like Receptor (TLR) signaling” and “NOD-like receptor (NLR) signaling” pathways (Figure 2). The *primed* microglia dark-green modules and the *acute* green-yellow module were significantly down-regulated in *primed* microglia mouse models and *acute* inflammation (LPS) compared to control. In the App-Ps1, Sod1, and LPS models, a significant enrichment for the “cellular metabolic process” GO category was observed. The App-Ps1 dark-green module was enriched for “neurotrophin” KEGG-pathway (p = 3.29E-5), suggesting reduced neuronal support by microglia in App-Ps1 mice (Figure 2). The *acute*, down-regulated green-yellow module was significantly enriched for the “lysosome” KEGG pathway (Figure 2), a category that was increased in *primed* microglia, further highlighting the fundamental differences between the *acute* classical M1-profile and the priming profiles. In addition, some model-specific differentially expressed modules were identified: the down-regulated brown module in physiological aging, which is enriched for “proteoglycan catabolic process” GO (p = 0.0023), the down-regulated magenta module in Ercc1, enriched for “cellular macromolecule metabolic process”- GO, and the dark-turquoise module which is up-regulated in all Sod1 samples, is unrelated to age of the animals, and is significantly associated with “cell-division” and “organelle organization” – GO’s (Figure 2c; p = 2.14E-6, and p = 1.46E-5 respectively). A complete list of all significantly enriched GOs and KEGGs is given in Additional file 7: Table S6.

### The priming modules strongly overlap with an independent age-induced microglia expression dataset

The effect of aging on microglia gene expression was recently determined by direct RNA sequencing with a focus on proteins for sensing endogenous ligands and microbes, referred to as the microglia sensome [36]. Using this dataset, genes significantly increased in expression during aging were determined and compared to our up-regulated *primed* and *acute* microglia modules. This microglia aging profile significantly overlapped with the *primed* microglia blue modules (ranging from p = 1.56E-25 to 6.39E-44; Figure 2a), and much less significant with the *acute* LPS-stimulated red module (p = 8.33E-13). This observation validates our observation that the up-regulated profiles of *primed* microglia is very similar to the transcriptional profile reported for aged microglia using an independent expression dataset.

### M1- and M2-classifications in relation to the blue and red modules

Microglia, in analogy to macrophage activation terminology, are often classified as M1 or M2, with M1 considered as a classical pro-inflammatory activation state and M2 as a tissue supportive, remodeling or anti-inflammatory state [10]. Using the WGCNA function userListEnrichment, the up-regulated *primed* blue and *acute* red modules were compared to M1 and M2 macrophage datasets (Figure 2). The *acute* red microglia module showed a highly significant overlap with LPS-stimulated macrophages (p = 2.45E-45), and this was a much more significant overlap than was observed with *primed* microglia (p = 1.22E-5 to 3.06E-16). Only the Sod1 up-regulated blue module had a minor overlap with the M2 up-regulated profile (p = 6.92E-5). These results suggested that the *primed* microglia phenotypes did not resemble a clear M1, M2 or intermediate phenotype.

### Microglia activation and priming is associated with a decreased expression of the ‘microglia unique signature’

Recently, a ‘microglia unique’ gene expression signature was reported [37], but the relationship between this signature and microglial activation is unknown. We compared this profile to our differentially expressed microglia modules. Where no significant overlap with the up-regulated blue module was detected, surprisingly all down-regulated *primed* dark-green and *acute* green-yellow modules significantly overlapped with this core microglial signature (Figure 2b; p-values ranging from p = 1.17E-5 to p = 8.11E-24). Hub genes of the down-regulated dark-green and green-yellow module were determined, and many of them were present in the microglia-unique expression signature, including *Mertk*, *Tmem119*, *P2ry12*, *P2ry13*, *SPARC,* and *Cx3cr1* (Additional file 8: Figure S2). This suggests that upon activation or priming, microglia not only acquire an activation signature, but also decrease their ‘surveilling’ state expression profile. The genes of down-regulated consensus modules are listed in Additional file 6: Table S5.

### Priming and acute LPS activation induce distinct transcriptional programs

**Figure 3:**
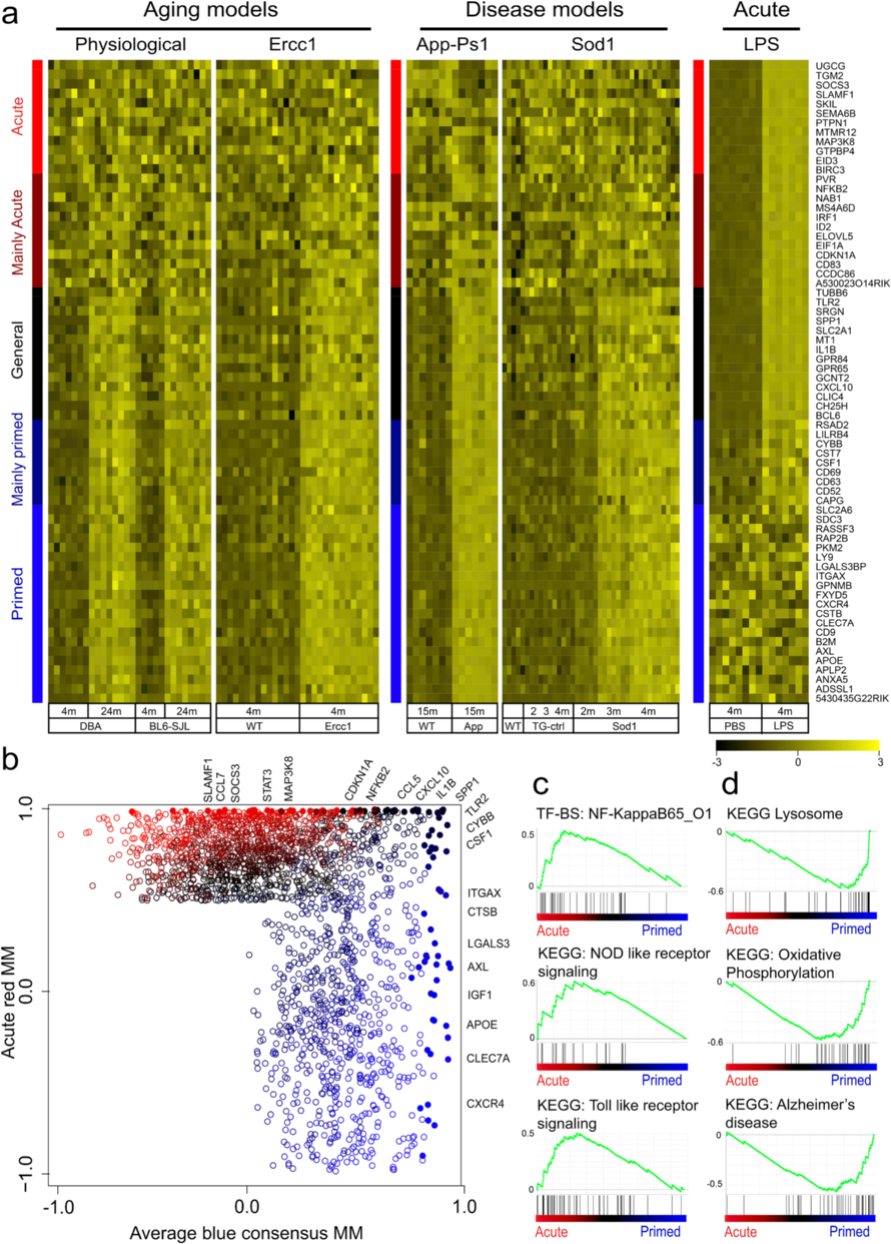
(Dis)similarities in *primed* and *acute*ly activated pure microglia. **a)** Heatmaps of *acute* to chronic activation categories. Hub genes (or genes that are centrally located in a module) were assigned by module membership. The 35 most correlated genes from the *acute* red and *primed* microglia blue module were categorized in 5 categories and depicted using a sidebar. “Acute-unique” hubs (red sidebar), “Mainly Acute” hubs with (lower) significant association in any of the *primed* models (dark-red sidebar), “General” hubs (black sidebar), “Mainly primed” hubs (dark blue sidebar) and “Primed” unique hubs (blue sidebar). **b)** Scatterplot of priming blue and *acute*ly activated red module membership values. For each gene which was significantly associated to either the *acute* or the *primed* networks, module membership values for the *acute* red and *primed* blue modules were plotted. Top 50 most connected hubs genes from *acute* and consensus *primed* microglia networks were assigned one of the five colors as described under a (filled dots). c, d) Gene set enrichment analysis of *primed* and *acute*ly activated microglia. **c)** *Acute* activated microglia were significantly enriched for NF-?B, NOD, and TLR signaling, **d)** *Primed* microglia were enriched for KEGGs lysosome, oxidative stress, and AD-signaling.

To determine the differences between *acute* activation and *priming*, the blue and red modules were compared using two approaches: hub gene classifications and ranked gene set enrichment analysis. First, WGCNA was used to identify hub genes that have a strong interrelation (i.e., an expression pattern highly similar to the module Eigengene (kME)). Hub genes have been reported to function as important determinants of a phenotype, for example as markers for cell types or intracellular biological processes [16]. For both the *acute* red and the consensus *primed* microglia blue modules, hub genes were determined. The 35 most ‘connective’ genes of both networks were categorized based on correlation thresholds (see materials and methods) in 5 groups according to the strength of the respective association with the up-regulated *acute* red and *primed* blue modules: “acute”, “mainly acute”, “general”, “mainly primed” and “primed” hubs and depicted as heat-maps (Figure 3a) and as a scatterplot (Figure 3b).

“Acute” hub genes mark processes specifically activated in the *acute* microglia response, whereas “primed” hub genes mark processes occurring in *primed* microglia. “General” hubs mark processes common to both *acute* and *primed* microglia and therefore relate to general microglial activation. Genes that belong to the “acute” hub category are not significantly associated with any of the other datasets and examples of “acute”-unique hub genes are *Map3k8* and *Socs3*. The “general” hub category contained genes that were up-regulated (and highly connected) in all five mouse models. This hub included genes like *Tlr2*, *Il-1*β, *Cxcl10,* and *Spp1,* representing a group of genes consistently up-regulated in activated microglia. Importantly, also a “primed” hub was identified containing genes specifically increased in expression and highly connected in *primed* microglia, including genes as *Apoe*, *Axl*, *Clec7a*, *Itgax* (also known as *CD11c*), and *Lgals3* (also known as *Galectin-3* and *Mac2*). Of these genes *Lgals3* has been associated with microglia priming during accelerated aging [10] and microglia activation following axonal injury [38]. Also two intermediate hub categories were defined, containing genes that were primarily highly connective in either *primed* microglia or *acute* LPS activation. The “mainly primed” hub contained genes like *Cybb* and *Csf1 (*also known as *Mcsf)* that were highly connected in the priming datasets and were also significantly correlated in the acute data set, but to a lesser extent. In the “mainly acute” hub, genes were significantly but not very strongly associated to any dataset other than the “acute” profile. It contained genes like *Nf-kb2* and *Irf1* that were highly connected in the *acute* dataset and were also significantly correlated in the *primed* data sets, although not in all mouse models or to a lesser degree.

Ranked gene set enrichment analysis was applied to determine potential functional differences between *primed* and *acute* networks directly. Gene sets that were significantly enriched in *acute* activation were NF-κB factor p65 (RelA), toll like receptor, and NOD like receptor signaling (Figure 2c). Gene sets significantly enriched in *primed* microglia were: Alzheimer’s and Parkinson’s disease signaling, oxidative phosphorylation, mitochondria, and lysosome (Figure 2d; for all annotations see Additional file 7: Table S6). These data indicate that the expression profiles of *primed* and *acute* activated microglia differed in several ways, and the most prominent changes were oxidative phosphorylation, and lysosome in *primed* microglia and NF-κB signaling in *acute* activation. These results are in agreement with the findings of the Webgestalt-KEGG-pathway analysis, further strengthening the notion that the *primed* microglia profile substantially differs from the M1 or M2-phenotype observed in *acute* activated microglia.

### Specific expression profiles for aged, Ercc1, App-Ps1, and Sod1 microglia

**Figure 4:**
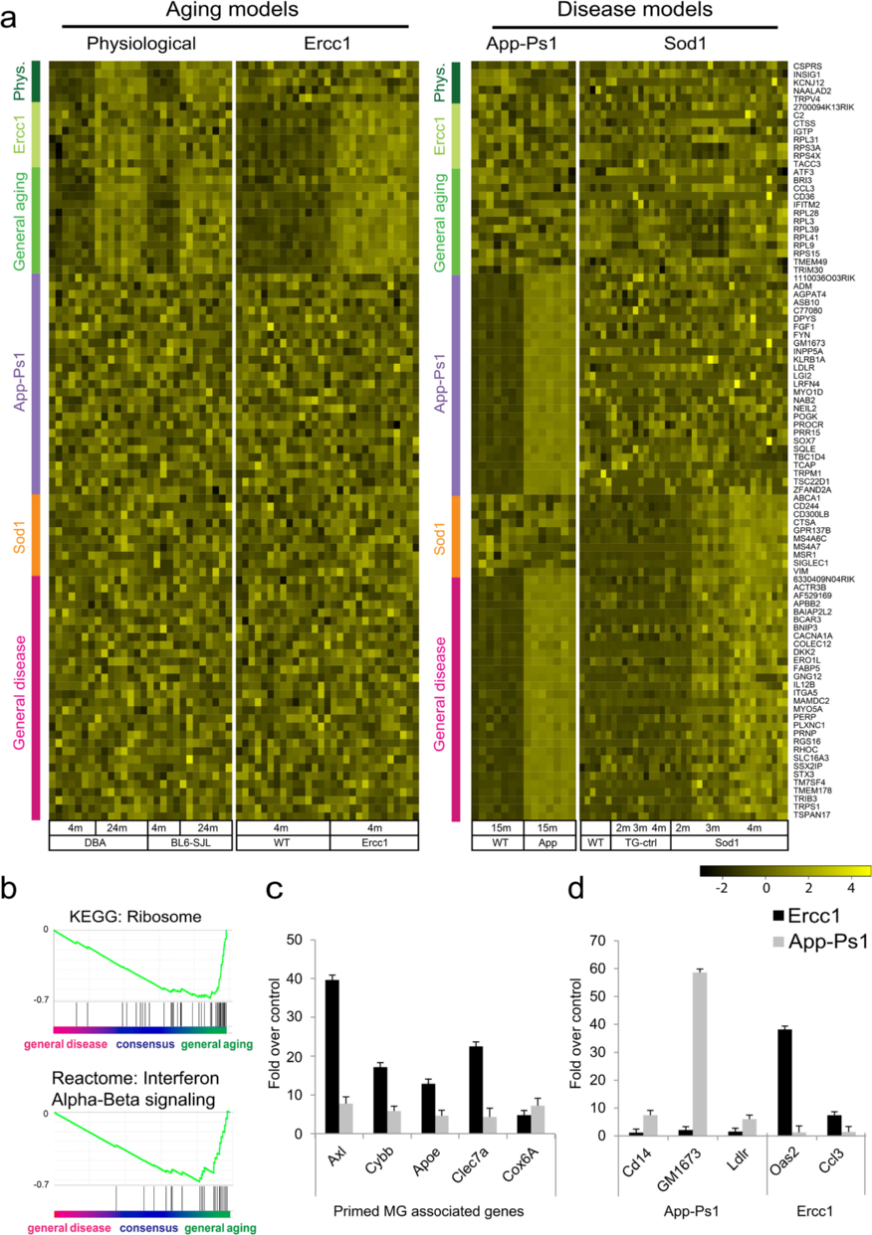
Model system-specific microglia transcriptional signatures. **a)** Heatmap of model system-specific hub genes. The top 100 most significantly associated module membership-genes from the App-Ps1, Sod1, Aged, and Ercc1 blue modules. Genes belonging to the consensus network were removed. The other genes were grouped according to significant association with both Aging models (green sidebar), both Disease models (dark-pink sidebar), physiological aging (dark-green sidebar), Ercc1 (dark-olivegreen sidebar), App-Ps1 (purple sidebar), and Sod1 (orange sidebar). **b)** Quantitative RT-PCR validation of consensus hub genes. RNA was isolated from App-Ps1 and Ercc1 FACS sorted microglia and mRNA expression levels were determined. The fold expression, normalized to HMBS, compared to its control with the standard error is depicted. **c)** Quantitative RT-PCR validation of model-specific hub genes. RNA was isolated from App-Ps1 and Ercc1 FACS sorted microglia and mRNA expression levels were determined. The fold expression, normalized to HMBS, compared to its control with the standard error is depicted. **d)** General aging microglia are significantly enriched for KEGG ribosome and IFNa-ß signaling. GSEA was used to determine differences between consensus chronic activation and *acute* activation. *Primed* microglia are enriched for KEGGs ribosome and IFNa-ß signaling.

As described above, a core consensus expression profile was found that describes the commonality of the *primed* microglia response in different mouse models. To determine mouse model-specific components, genes that significantly associated to the blue module in any, but not all of the mouse models were selected. These genes were grouped according to specificity and association strength 1) to the individual mouse models, 2) to both aging models (physiological aging + Ercc1 accelerated aging), or 3) to both neurodegenerative disease models (App-Ps1 + Sod1; Figure 4a). To functionally annotate the differences between conditions, ranked gene set enrichment analysis was performed for aging models (aging + Ercc1) vs. disease models (App-Ps1 + Sod1). Gene sets significantly enriched in aging models were related to ribosome activity and interferon alpha/beta signaling (Figure 3b). The aging specific ribosome activity was supported by ribosome-related hub genes RPL3,9,28,39,41 and RPS15. No gene sets were significantly enriched in the general neurodegeneration or individual neurodegeneration disease modules. The genes of these model-specific modules are listed in Additional file 6: Table S5.

### Validation of the primed blue module and model system-specific gene profiles

Differential expression of several hub genes of the consensus blue module as well as mouse model-specific gene expression was validated using quantitative RT-PCR analysis of Ercc1 and App-Ps1 microglia. Expression levels of *primed* microglia blue module hub-genes *Axl, Cybb, Apoe, Clec7a,* and *Cox6a* were determined (Figure 3b). All these genes were significantly increased in Ercc1 and App-PsS1 microglia compared to controls, confirming the validity of the consensus *primed* microglia blue module. *Lgals3* is a hub gene in the *primed* microglia consensus module and identified as a marker for *primed* microglia in accelerated aging Ercc1 mutant mice [39]. Brain sections of aged, Ercc1, and App-Ps1 mice were stained and Iba1/Lgals3 double positive cells were only observed in aged, Ercc1, and App-Ps1 animals and not in young or aged-matched controls (Additional file 9: Figure S3). Mouse model-specific expression of several hub genes was confirmed using quantitative RT-PCR analysis of Ercc1 and App-Ps1 microglia. *CD14*, *Gm1673,* and *Ldlr* expression was restricted to the App-Ps1 blue module and significantly induced in 15 months old App-Ps1 microglia while their expression was not increased in Ercc1 microglia (Figure 3d). The *Ccl3* gene was restricted to the general aging module and the *Oas2* gene was most significantly associated with the Ercc1 blue module. Their expression level was significantly increased in Ercc1, but not in App1-Ps1 microglia, confirming the specificity of the identified sub-modules.

### Signatures of acute and primed microglia are preserved in brain tissue samples

To determine, if the transcriptional profiles associated with *primed* microglia are preserved in mouse brain tissue, expression sets of App-Ps1, aged, rTg4510 and ME7 prion infected mice (-/+ LPS) were analyzed. rTg4510 mice overexpress the a mutant form of human tau that causes fronto-temporal dementia and parkinsonism linked to chromosome 17 (FTDP-17). rTg4510 mice provide a model for tauopathies, including Alzheimer’s disease. ME7 prion brain infection is a frequently used model system to induce microglia priming. The overlap between the up-regulated blue modules in pure microglia and significantly up-regulated genes in brain tissue expression data was determined as a measure of preservation. Significant overlap was observed between pure microglia up-regulated blue modules and the up-regulated genes in *App-Ps1* (p-values ranging from 7.35E-10 to 2.68E-32) and aging (p-values ranging from 9.24E-7 to 7.56E-20; Additional file 10: Table S7) brain tissue. No significant overlap of the upregulated App-Ps1 and aging genes was observed with the up-regulated *acute* red module of microglia from LPS injected mice. Interestingly, the ME7 response genes (main effect of ME7) significantly overlapped with the *primed* blue modules (p-values ranging from 7.31E-17 to 2.58E-50), but much less with the *acute* red module (p = 3.5E-9). In contrast, a highly significant overlap between the LPS response genes (main effect of LPS) with the up-regulated *acute* red module was observed (Additional file 10: Table S7, p = 8.7E-36) and the overlap with the blue *primed* modules was less pronounced (p-values ranging from 1.67E-4 to 1.74E-14). Similar results were obtained with the rTg4510 dataset; a highly significant overlap with the *primed* blue microglia modules was found (p-values ranging from 5.95E-8 to 2.68E-30). These data suggest that signatures of *primed*, but not *acute* activated, microglia are preserved in brain tissue expression data from models of Alzheimer’s disease, prion infection and, aging.

### Comparative WGCNA analysis of brain tissue and pure microglia expression data

**Figure 5:**
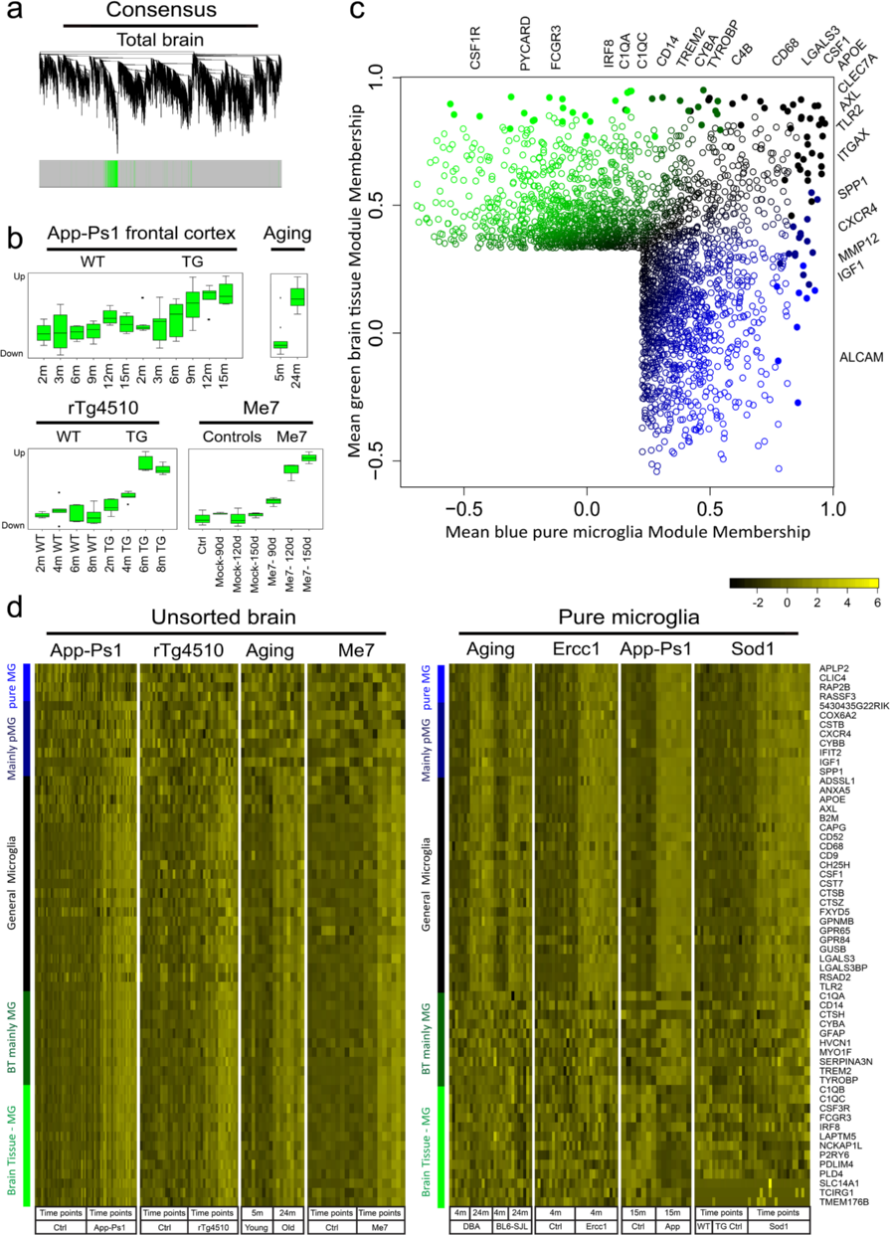
Comparison of pure-microglia and brain tissue-derived microglia-enriched modules. **a)** Consensus co-expression networks of brain tissue transcriptomes. Co-expression networks were generated for the indicated brain tissue transcriptome datasets. **b)** brain tissue consensus module expression boxplots. The ME of the green microglia-enriched module in the Me7, App-Ps1, Aging, and rTg4510 datasets was significantly up-regulated in all model systems. ME expression across all datasets and conditions is depicted as box-and-whisker plots. **c)** Scatterplot of the hub genes of the consensus pure microglia module and the brain tissue microglia-enriched module. For each gene, which was significantly associated to the pure microglia or the brain tissue microglia-enriched networks, module membership values for the brain tissue green and microglia modules were plotted. Top-50 most connected hubs genes from brain tissue to pure microglia were assigned one of five colors (filled dots). “Pure microglia” (blue), “Mainly pure microglia“ (dark-blue), “General microglia” (black), “Mainly brain tissue-derived microglia signature” (dark-green) and “brain tissue-derived microglia signature” (green). **d)** Heatmaps of pure microglia to brain tissue microglia-enriched categories. Heatmaps of the consensus profiles of pure microglia and brain tissue datasets as indicated are depicted. Hub genes were assigned by module membership, the top-35 most correlated genes from the pure microglia and brain tissue microglia-enriched consensus modules were categorized in 5 categories as described in c).

WGCNA has successfully been applied to brain tissue expression data to identify modules enriched for particular cell types like microglia [1,17,36,40], but it is currently unclear to which degree these microglial modules resemble the profile of pure microglia. We applied WGCNA to brain tissue (App-Ps1, aged, Me7, and rTg4510) and pure microglia (aged, App-Ps1 and Ercc1) datasets, to generate two parallel consensus networks (Figure 5a). In brain tissue expression data, a consensus green module was found, that is significantly up-regulated with aging and neurodegeneration (Figure 4b). The brain tissue consensus green WGCNA module significantly overlapped with microglia modules reported in other brain tissue expression studies (p = 1.19E-57 to 8.41E-32; Additional file 11: Table S8a).

The overlap between the consensus brain tissue green module with the identified individual pure microglia blue modules, the consensus blue module, and the *acute* LPS red module was determined (Additional file 11: Table S8b). A significant overlap was observed with all primed microglia blue modules (p = 5.16E-48 to 4.95E-18) but a less significant overlap was present with the *acute* activation red module (1.61E-7). Next, this consensus green microglia-enriched profile was compared to the consensus blue *primed* microglia profile (Figure 1a), hub genes were allocated, and five categories were defined (see Methods): “brain tissue derived microglia signature”, “mainly brain tissue derived microglia signature”, “general microglia”, “mainly pure microglia”, and “pure microglia”. The “general microglia”-hub consists of highly connective genes that were found both in the pure microglia and in the microglia-enriched brain tissue modules and contains genes like: *Spp1, Csf1, Axl, B2m, Lgals3bp,* and *Tlr2*. The “pure microglia” hub contains, amongst others, the *Clic4, Rap2B,* and *Gapdh* genes that are highly connective in microglia but not in the microglia-enriched brain tissue module. The “brain tissue-derived microglia signature” hub contains genes like *C1QB, C1QC,* and *Irf8*. The intermediate “mainly pure microglia” hub contains genes like *Cybb* and *Igf1*, and the “mainly brain tissue microglia” hub contains previously reported microglial hub genes *Tyrobp* and *Trem2,* as well as the astrocyte marker *Gfap* (Figure 4c,d). The genes of these pure microglia and brain tissue modules are listed in Additional file 6: Table S5. Since these data indicate that C1QB, C1QC, Tyrobp and Trem2 expression is not increased in *primed* or *acute* activated microglia, the expression level of these genes was checked a recently published database by Zhang and colleagues [41] and we found that all these genes are very highly expressed in microglia and therefore likely identified as hub genes for microglia in brain tissue expression data.

These data show that the consensus profile of microglia (-enriched) modules found in different pure microglia and brain tissue expression datasets share similarities at the hub gene level, but also are critically different. These differences are likely caused by a combination of changes in microglia cell numbers in the brain under neuropathological conditions and microglia-intrinsic changes in gene expression. Several papers have shown that neuropathology is associated with increased microglia cell proliferation [10,24,42]*.* As a consequence, typical hub genes of microglia modules identified using brain tissue expression data are not necessarily hub genes in pure microglia expression data.

## Discussion and conclusions

**Figure 6:**
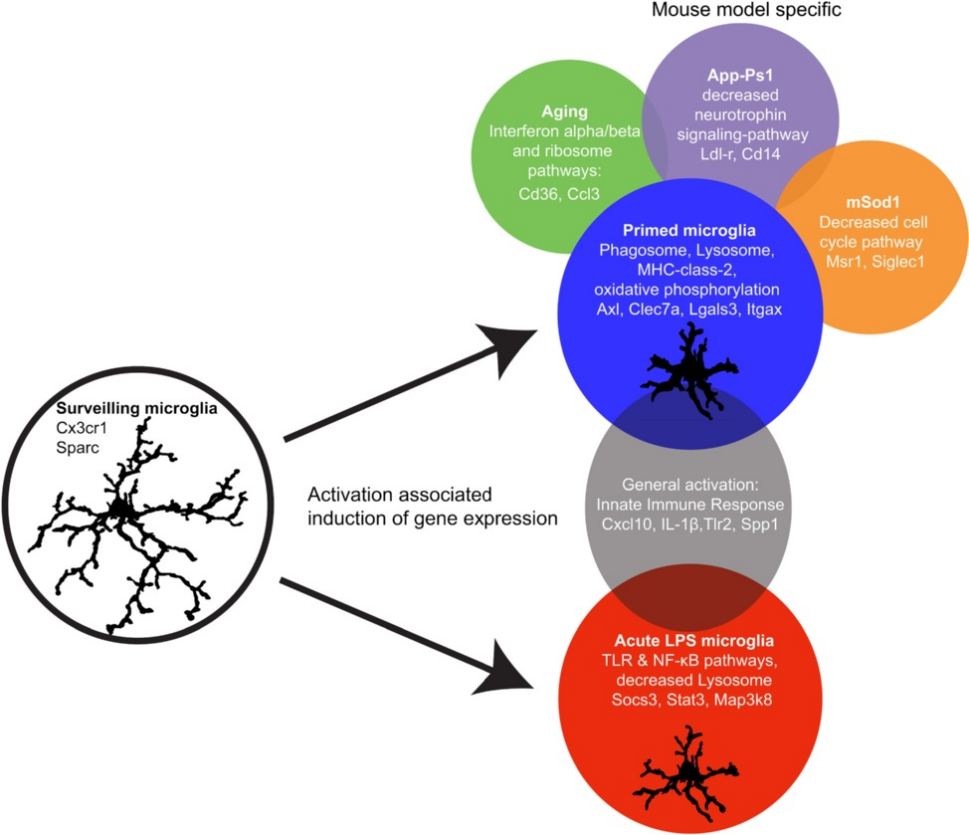
Summary figure describing the main findings of the current paper. Surveilling microglia are activated either acutely by a ligand such as LPS or by a neurodegenerative and aging brain environment.

*Primed* microglia are characterized by hypersensitive responses to proinflammatory stimuli. It has been suggested that priming of microglia is induced by chronic exposure to low-grade inflammation, as observed in neurodegenerative diseases and brain aging [9]. Microglia priming has been described to occur during aging and in a variety of CNS-diseases including AD, Parkinson’s disease, Multiple Sclerosis, ALS, stroke, Wallerian degeneration, and Me7 prion infection [43]. Furthermore, it is hypothesized that this hyper exaggerated responsiveness of the *primed* microglia contributes to the observed neurodegeneration [11]. The signaling pathways and mechanisms involved in the induction of priming are unknown. We therefore set out further to characterize the mechanisms of microglia priming using gene expression profiling in mouse models for aging and neurodegenerative disease. Using WGCNA we have identified specific gene expression networks associated with microglia priming. A visual summary of the main findings of this manuscript are depicted in Figure 6.

One of the main objectives of this study was to investigate the hallmarks of gene expression profiles of *primed* microglia isolated from mouse models for aging and neurodegeneration. We show that in all mouse models investigated, independent of the origin and platform used, these *primed* microglia expressed a core gene expression profile, which substantially differed from the inflammatory gene network observed in *acute*ly activated, pro-inflammatory, microglia. The degree of preservation of this core gene expression profile in physiological aging, Ercc1, App-Ps1, Sod1, and Me7 mice made it very likely that these microglia acquired a similarly *primed* phenotype. In the current paper, we show that *primed* microglia are clearly different from M1 and M2 macrophages or M1 ex-vivo isolated microglia. The observation that activated microglia in chronic brain disease/in a neurodegenerative disease do not resemble an M1 or M2 phenotype was already suggested by Chiu et al. [4], whose Sod1-dataset was used in the current study. They proposed that the pattern of regulation of a particular set of genes, including Axl, can distinguish LPS stimulated microglia from Sod1-associated microglia. Based on the *primed* microglia gene expression network we predict that that *primed* microglia are characterized by expression of cell surface markers like *Itgax, Lgals3, Axl, Clec7a, MHC class 2,* and *Cxcr4*.

The major functions of the *primed* microglia gene expression network show that these cells are involved in immune-, phagosome-, lysosome-, oxidative phosphorylation, and antigen presentation signaling pathways. These functions fit the needs of chronically degenerating brain tissue. In response to tissue damage, microglia migrate to the site of injury and phagocytose tissue debris or damaged cells [6], thereby potentially degrading healthy synapses and contributing to the ongoing degenerative process [44]. In the used mouse models for neurodegeneration and aging, the phagosome and closely related lysosome KEGG pathways were indeed significantly enriched in the consensus blue module, suggesting aging- or neuropathology-induced phagocytic activity of *primed* microglia.

Using WGCNA, hub genes were identified that are likely candidate genes that drive the observed phenotype [1,19-21]. Interestingly, four hub genes unique to the *primed* microglia gene expression network, *Galactin-3*, *Igf1, Csf1*, and *Axl* were previously shown to be instrumental for microglia functions including proliferation, activation, and phagocytosis. *Igf1* signals through *Galactin-3* and inactivation of the *Galactin-3* gene resulted in *Igf1* insensitivity, decreased microglia activity and a significant increase in the ischemic lesion size [38,45]. *Igf1* signaling is related to neuroplasticity and neuroprotection [46] and is shown to mediate motor neuron protection and prolonged survival in ALS animal models[4]. Csf-1-mediated microglia proliferation has been shown to be important in chronic neurodegeneration [41] and inhibition of Csf-r1 signaling in mice resulted in complete ablation of microglia [47]. The tyrosine kinase receptor *Axl* is up-regulated in microglia in various neurodegenerative and demyelinating conditions, such as cuprizone-induced demyelination, EAE and in MS lesions, and is shown to play an important role in phagocytosis of apoptotic cells and myelin [48-50]. *Axl* KO mice experience enhanced inflammation and delayed myelin removal in EAE-mice [49], and fewer mature oligodendrocytes and more axonal damage in cuprizone induced demyelination [48]. Contrarily, a recent paper showed that Axl is an inflammatory phagocytic receptor whose expression was induced by pro-inflammatory mediators [51]. The up-regulation of these hub-genes suggests that microglial priming also has adaptive aspects, necessary to cope with increased neurodegeneration and environmental stress.

In a recent study, microglia were compared to other myeloid immune cells and a microglial signature, which is dependent on *TGF*β signaling, was reported [38]. This list of genes was enriched in the down-regulated modules of both *primed* and *acute*ly activated microglia. Two genes that are particularly interesting in this respect are *SPARC* and *Cx3cr1*, which are hub genes of the down-regulated consensus module. *SPARC* regulates the activity of growth factors and cytokines. Enhanced microglia proliferation, microgliosis around stroke lesions, and enhanced recovery is observed in SPARC null mice [52]. *Cx3cr1* is ubiquitously expressed by microglia and plays an important role in microglia-neuron communication [53]. It was shown that *Cx3cr1* deficiency resulted in microglia activation, and increased neurodegeneration following LPS injections in PD and ALS-models [49]. Moreover, *Cx3cr1* deficiency worsens the AD-related neuronal deficits, associated with microglial activation and elevated chemokines [50]. In contrast, others reported that in two mouse models for AD, *Cx3cr1*-deficiency resulted in increased beta amyloid clearance and prevented neuron loss [54,55]. Furthermore, lack of *Cx3cr1* was shown to reduce infarct size, ischemic damage and inflammation [56]. The notion is that constitutively expressed *Cx3cl1* (the ligand for Cx3cr1) provides a tonic inhibitory signal to microglia to remain quiescent, and that deficiency results in hyperactivated microglia [57,58]. This indicates that upon activation, microglia partially lose their resting signature and acquire a *priming* or *acute* signature.

Besides the aforementioned common *primed* microglia gene expression network, additional, specific elements of the microglia gene expression networks were found, exclusively for the aging, AD, or ALS mouse models used in our study. Although it is impossible to eliminate potential confounding factors like isolation protocols, mouse strain, age, CNS regions used, and different expression profiling methodologies, we could confirm the model-specific differences in gene expression in App-Ps1 and Ercc1 mice with quantitative RT-PCR. These model-specific changes in gene expression were related to an increased interferon-type 1 signature in both aging models, an altered cell-cycle GO in Sod1 and decreased neurotrophin signaling in App-Ps1. An aging-associated type-1 interferon signature is recently described in the choroid plexus of aging mice and humans [59]. This interferon signature had a negative effect on brain function, and was induced by brain-derived signals derived from the CSF. In addition, it was suggested that interferon type 1 plays a role in microglial priming [42].

Microglia isolated from App-Ps1 mice were hallmarked by a decreased expression of neurotrophin related genes. In App-Ps1 mice, amyloid plaque load increases with age and is associated with a strong immune signaling profile to which microglia contribute [60]. Interestingly, two App-Ps1 model system-specific hub-genes, *LDL*-receptor and *CD14*, are associated with Amyloid Beta (Aβ) clearance. Microglia surround amyloid fibril deposits and have been suggested to be involved in their phagocytosis [61]. Increased *LDL* receptor expression prevented amyloid deposition and led to an increased Aβ clearance [62]. CD14 is required for Aβ stimulation of microglia and inhibition of CD14 prevents initiation of Aβ phagocytosis [62]. This indicates that using WGCNA, we could identify disease-specific module components with known biological relevance in AD.

WGCNA is often used to generate microglia specific profiles in brain tissue expression data [1,19,21]. Similarly, we identified a microglia-enriched consensus module in brain datasets, indicating that *primed* microglia in Me7, aging, rTg4510, and App-Ps1 mice had a similar gene expression profile. However, comparison of this brain tissue microglia-enriched module to the pure microglia expression profiles, showed substantial differences in hub genes. In the brain tissue data, *Trem2* and *Tyrobp* are identified as hub genes, where in pure microglia they are only weakly associated with the consensus *priming* module, suggesting that these genes might not play a critical role in microglia *priming*. Tyrobp and Trem2 are highly expressed in microglia, and therefore often identified as hub genes of microglial modules in brain tissue datasets. Changes caused by altered relative cell numbers in brain tissue expression data are readily detected using WGCNA, resulting in cell type-specific modules. However, it is very difficult to discriminate between cell intrinsic alterations in gene expression levels and changes in cell numbers in cell type-specific modules. In addition, other cell types, such as astrocytes, possibly contaminate these modules. Summarizing, our data indicate that a complementary WGCNA analysis of both pure cell populations and brain tissue expression data is required in order to fully unveil regulatory gene networks.

In this study, we analyzed *primed* microglia from different neurodegenerative conditions. Microglia *priming* is often regarded as a confounding factor, resulting in exacerbation of neurodegeneration in a wide range of conditions [11]. The core microglia *priming* module described in this study supports the notion of a generic microglia response in different neuropathologies, but this module mostly contains genes related to phagocytosis and cell proliferation, with tissue protective elements. This indicates that *primed* microglia adopt an altered inflammatory profile predominantly adapted for phagocytic clearance and in a state of immune readiness, possibly necessary to cope with inflammatory and neurodegenerative conditions.

## Additional files

Additional file 1: Table S1. Overview datasets and references included in the meta-analysis. Listed datasets were obtained via GEO or Arrayexpress or generated by either of the authors from the current manuscript and used in this manuscript. Additional file 2: Table S2. Primer list of qPCR primers used for validation of hub-genes. Additional file 3: Table S3. Differential gene expression analysis. In addition to the WGCNA analysis the genes included in the analysis differential gene expression analysis was performed and the results are depicted. Additional file 4: Table S4. Differential Module Eigengene (ME) expression for all modules of all pure microglia datasets. Kruskall Wallis between group test was applied to determine whether a module was differentially expressed or not. In addition the direction of differential expression is described as up- or down regulated with genotype or phenotype. Additional file 5: Figure S1. Overlap heatmap. Differentially expressed modules were compared using a Fisher’s exact test and depicted as a heatmap in which the intensity of the red color corresponds to p-value. A p-value cut-off of 1E-100 was used. Additional file 6: Table S5. Genes module assignments, module membership and mouse model specific elements. WGCNA was applied to the expression datasets and genes were assigned to modules using a hard-clustering approach as well as with Module Membership values with corresponding FDR multiple testing corrected p-values. Additional file 7: Table S6. Annotations - Results of WEBGESTALT and GSEA enrichment analyses. WEBGESTALT GOs, KEGGs and Benjamini Hochberg Multiple Testing corrected p-values for all significantly differentially expressed modules. Additional file 8: Figure S2. (Dis)similarities in the down-regulated *primed* microglia and *acute* activated microglia modules. a) Heatmaps of *acute* to *primed* categories. Hub genes were assigned by module membership. The top-35 most correlated genes from the *acute* green-yellow and primed dark-green module were categorized in 5 categories and depicted using a sidebar. Acute-unique hubs (green-yellow sidebar), Mainly Acute hubs with (lower) significant association in any of the chronic models (yellow sidebar), General hubs (grey sidebar), Mainly primed hubs (green sidebar) and Primed unique hubs (dark-green sidebar). b) Scatterplot of consensus *primed* microglia dark-green and *acute* green-yellow module memberships- values. Module membership values of the consensus priming dark-green and *acute* green-yellow down-regulated modules were depicted as a scatterplot. In addition, highlighted genes are present in the recently reported microglia core signature [38], which is enriched in the down-regulated profiles. Additional file 9: Figure S3. Primed microglia in aged, Ercc1 and App-Psn1 mice. Staining of *primed* microglia in i.p. PBS or LPS injected, aged, Ercc1, and App-Ps1 mice. Coronal (App-Ps1) and sagittal (rest) sections were co-stained with Iba1 (microglia, green) and Lgals3 (or Mac2) (*primed* microglia, red). Representative images of the cortex and brain stem are depicted. No Mac2 expression was detected in LPS injected mice, *primed* microglia were detected in both cortex and brain stem in aged mice (indicated by arrows). As previously reported [10], in *Ercc1* mutant mice, microglia *priming* was most pronounced in the brain stem (arrow). In App-Ps1 animals, plaque-associated microglia also expressed Mac2 (arrow), the brain stem was present in the sections analyzed. Additional file 10: Table S7. Preservation of the chronic microglia module brain tissue datasets. Differentially expressed gene lists from brain tissue datasets: App-Ps1 and control frontal cortex, rTg4510 and control brain tissue, aging brain tissue, ME7 inoculation hippocampus with and without LPS and ME7 and mock inoculation on different time points were used as input for userlistenrichment function. Significance of the overlap between the significantly differentially expressed gene lists and the blue and red (up-regulated) modules is depicted. Additional file 11: Table S8. Overlap between brain tissue and pure microglia priming microglia modules. a) The significance of the overlap between the green consensus brain tissue microglia module and other microglia modules identified in brain tissue datasets is depicted. b) The significance of the overlap between the green consensus brain tissue microglia module and the pure microglia priming blue modules and acute activated red module is depicted.
