# Supplementary figures and images for: Induction of a common microglia gene expression signature by aging and neurodegenerative conditions: a co-expression meta-analysis
Source: Acta Neuropathol Commun. 2015 May 23;3:31. doi: 10.1186/s40478-015-0203-5 (PMC4489356; doi:10.1186/s40478-015-0203-5)

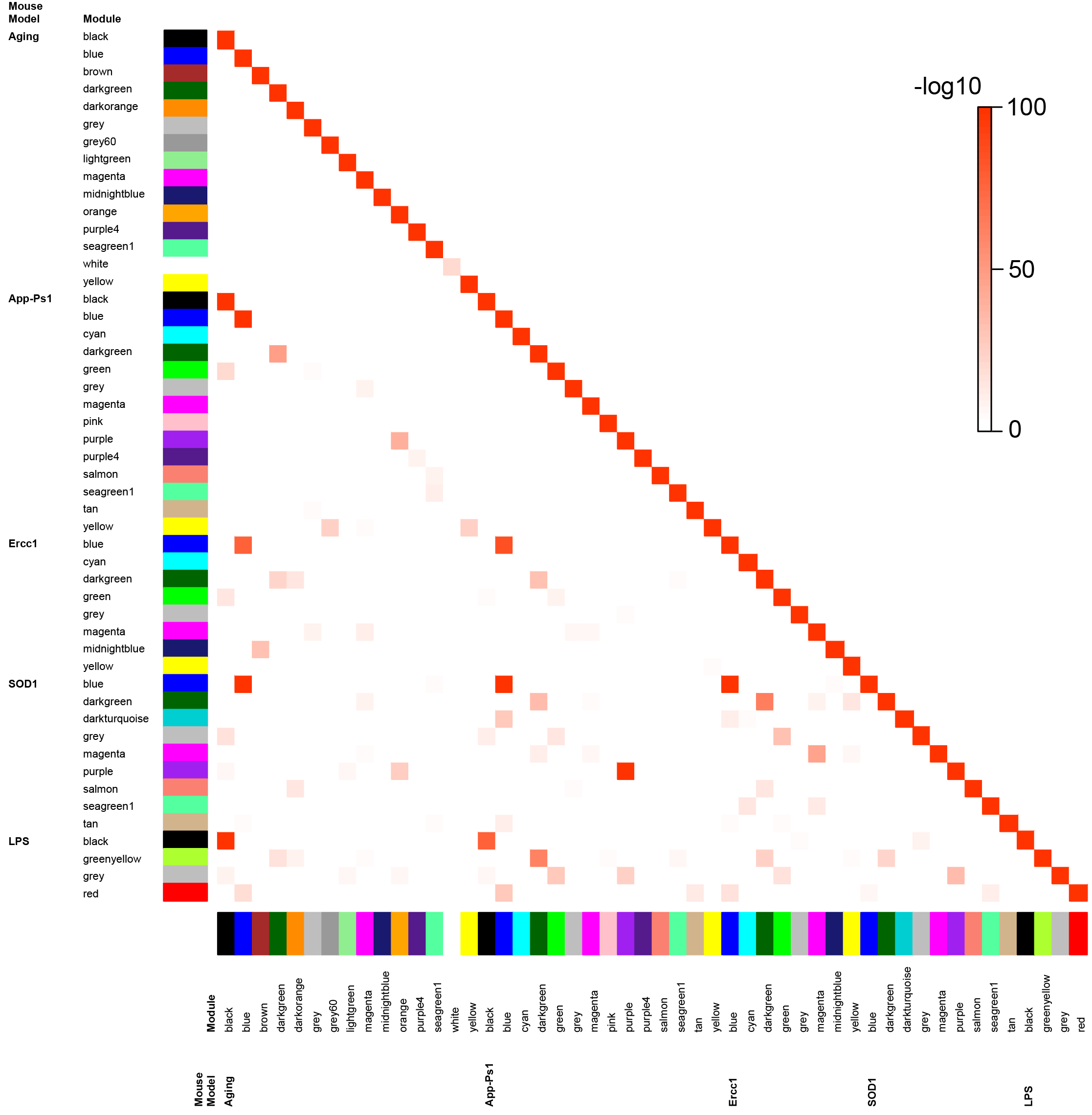

Supplement: Additional file 5: Figure S1. — Overlap heatmap. Differentially expressed modules were compared using a Fisher’s exact test and depicted as a heatmap in which the intensity of the red color corresponds to p-value. A p-value cut-off of 1E-100 was used. [file 40478_2015_203_MOESM5_ESM.tif]

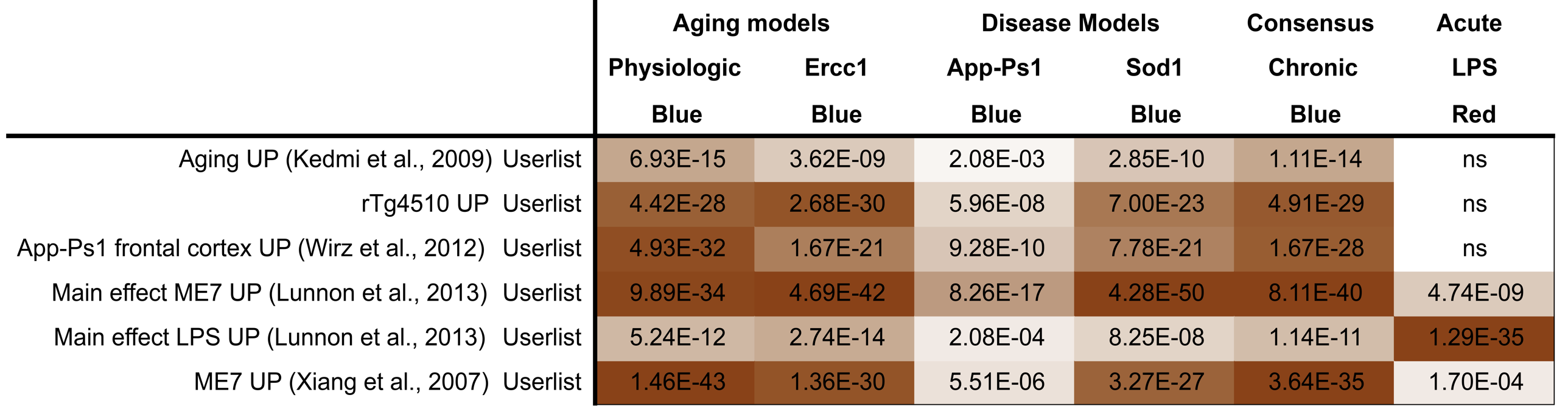

Supplement: Additional file 10: Table S7. — Preservation of the chronic microglia module brain tissue datasets. Differentially expressed gene lists from brain tissue datasets: App-Ps1 and control frontal cortex, rTg4510 and control brain tissue, aging brain tissue, ME7 inoculation hippocampus with and without LPS and ME7 and mock inoculation on different time points were used as input for userlistenrichment function. Significance of the overlap between the significantly differentially expressed gene lists and the blue and red (up-regulated) modules is depicted. [file 40478_2015_203_MOESM10_ESM.tif]

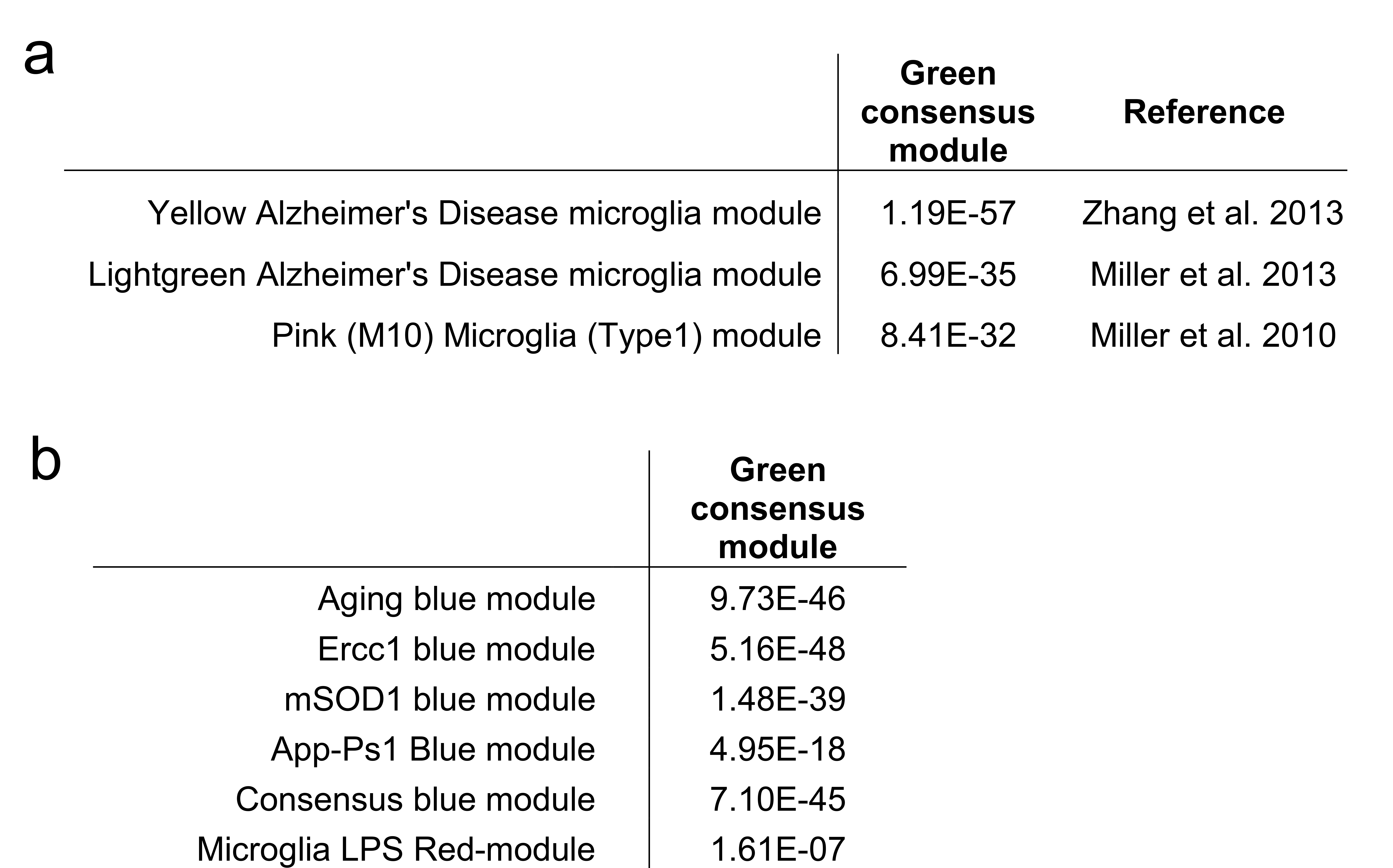

Supplement: Additional file 11: Table S8. — Overlap between brain tissue and pure microglia priming microglia modules. a) The significance of the overlap between the green consensus brain tissue microglia module and other microglia modules identified in brain tissue datasets is depicted. b) The significance of the overlap between the green consensus brain tissue microglia module and the pure microglia priming blue modules and acute activated red module is depicted. [file 40478_2015_203_MOESM11_ESM.tif]
